# *F8* gene inversion and duplication cause no obvious hemophilia A phenotype

**DOI:** 10.3389/fgene.2023.1098795

**Published:** 2023-02-09

**Authors:** Shaoying Li, Jianchun He, Liming Chu, Shuai Ren, Wenzhi He, Xiaoyan Ma, Yanchao Wang, Mincong Zhang, Lingyin Kong, Bo Liang, Qing Li

**Affiliations:** ^1^ Department of Obstetrics and Gynecology, Experimental Department of Institute of Obstetrics and Gynecology, The Third Affiliated Hospital of Guangzhou Medical University, Key Laboratory for Major Obstetric Diseases of Guangdong Province, Guangzhou, China; ^2^ Basecare Medical Device Co., Ltd, Suzhou, China; ^3^ State Key Laboratory of Microbial Metabolism, Joint International Research Laboratory of Metabolic and Developmental Sciences, School of Life Sciences and Biotechnology, Shanghai Jiao Tong University, Shanghai, China

**Keywords:** F8, hemophilia A, intron 22 inversion, F8 duplication, C1qA

## Abstract

Hemophilia A (HA, OMIM#306700) is an X-linked recessive bleeding disorder caused by the defects in the *F8* gene, which encodes coagulation factor VIII (FVIII). Intron 22 inversion (Inv22) is found in about 45% of patients with severe hemophilia A. Here, we reported a male without obvious hemophilia A phenotype but bearing an inherited segmental variant duplication encompassing *F8* as well as Inv22. The duplication was approximately 0.16 Mb and involved from exon 1 to intron 22 of *F8*. This partial duplication and Inv22 in *F8* was first found in the abortion tissue of his older sister with recurrent miscarriage. The genetic testing of his family revealed that his phenotypically normal older sister and mother also had this heterozygous Inv22 and a 0.16 Mb partial duplication of *F8*, while his father was genotypically normal. The integrity of the *F8* gene transcript was verified by sequencing of the adjacent exons at the inversion breakpoint, which explained why this male had no phenotype for hemophilia A. Interestingly, although he had no significant hemophilia A phenotype, the expression of *C1QA* in his mother, sister, and the male subject was only about half of that in his father and normal population. Our report broadens the mutation spectrum of *F8* inversion and duplication and its pathogenicity in hemophilia A.

## Introduction

Hemophilia A (coagulation factor VIII(FVIII) deficiency), which is caused by the mutation in *F8* gene and leads to abnormal production or function of FVIII protein, is the most common clinical hereditary hemorrhagic disease, with an incidence of about 1/5,000 in males (Lenting et al., 1998). *F8* gene locates at the end of the long arm of X chromosome (Xq28), including 25 introns and 26 exons, with a total length of about 186 kb (Gitschier et al., 1984; Freije and Schlessinger, 1992). Intron 22 contains a 9.5-kb sequence Int22h-1, which is about 5.78 kb downstream of exon 22, and there are two homologous repeats (Int22h-2 and Int22h-3) of about 497 kb upstream of *F8* gene. Intron 22 inversion (Inv22) is caused by this homologous recombination of Int22h-1 and Int22h-2/Int22h-3 (Lakich et al., 1993). A broad spectrum of mutations is known to cause hemophilia A, and Inv22 is the main pathogenic mechanism, accounting for about 45% of severe hemophilia A (Gouw et al., 2012; Lu et al., 2020). Large duplications of *F8* are relatively uncommon, compared to the small genetic variants, and account for approximately 0.07% (Lannoy and Hermans, 2016). *F8* duplications have varying clinical implications, from benign to severe HA phenotypes, depending on the location of insertion of the gained region (Zimmermann et al., 2010; Lannoy et al., 2013; Lannoy et al., 2015; Lannoy et al., 2019).

Here, we described a Chinese pedigree presented without hemophilia A but harboring partial duplication and Inv22 in *F8.* To the best of our knowledge, this is the first report of such complex structural variation of *F8*.

## Materials and methods

CNV analysis was based on low-depth whole-genome sequencing. CNV-Seq was conducted by using the DA8600 sequencer (Daan Gene), and tMAP (version 4.6), Picard (version 2.18.17), LOWESS regression, and circular binary segmentation were used for CNV analysis (Liu et al., 2020). Inv22 assay was performed using the *F8* specific inversion panel. Briefly, we performed PCR amplification by three specific primers (B: 5′- CCC​CAA​ACT​ATA​ACC​AGC​ACC​TTG​AAC​TTC​CCC​TCT​CAT​A-3’; P: 5′- GCC​CTG​CCT​GTC​CAT​TAC​ACT​GAT​GAC​ATT​ATG​CTG​AC-3’; and Q: 5′- GGC​CCT​ACA​ACC​ATT​CTG​CCT​TTC​ACT​TTC​AGT​GCA​ATA-3′). The P&Q combination could amplify a 12-kb amplification product in samples without Inv22, while the B&Q combination could amplify an 11-kb amplification product in samples with Inv22. Detailed experimental procedures were described previously (Mahmoud Abu Arra et al., 2020).

Total RNA was isolated from each blood sample of the family members, and RNA-seq was conducted using the Illumina NovaSeq platform with 2 × 150 bp pair-end reads. FastQC (v0.11.5) was used for quality control of raw data. Adaptor sequences were removed using Cutadapt (v3.0). The proportion of ribosomal RNA (rRNA) in reads was assessed using CollectRNASeqMetrics of GATK (v4.1.2.0).

Data were aligned to the human reference genome (GRCh37) using STAR (v2.7.7a) (Dobin et al., 2013). HTseq-count (v0.12.4) was used to extract the number of each gene reads, and DEGSeq was used for differential expression analysis. Criteria for differential expression gene screening were q-value ≤0.05 and log fold change ≥1. Reference databases for male and female samples were constructed as follows: sample IDs of whole-blood samples were obtained from the file (GTEx_Analysis_v8_Annotations_SampleAttributesDS.txt); gene reads of the corresponding samples were obtained from the file (GTEx_Analysis_2017–06–05_v8_RNASeQCv1.1.9_gene_reads.gct.gz); and gender information was obtained from the file (GTEx_Analysis_v8_Annotations_SubjectPhenotypesDS.txt). The median (refmedian) and mean (refmean) of each gene were taken as read counts of the corresponding gene in the reference database. The medians of ratios analyzed using DESeq2 were used to normalize each sample (Anders and Huber, 2010). The analysis of differentially expressed genes was obtained by comparing data from the male (II-4) with his father (I-2), older sister (II-2), mother (I-1), and the database (GTEx database (v8)). The values of the read counts after normalization using DESeq2 are shown in Supplementary Material S1, and the codes for RNA-seq data analysis are provided in Supplementary Material S2. RNA-seq data were deposited on the Genome Sequence Archive for Human (https://ngdc.cncb.ac.cn/gsa-human/) with accession number HRA003790, and data on chromosomal microarray analysis were deposited on OMIX (https://ngdc.cncb.ac.cn/omix/) with accession number OMIX002784.

## Results

### Patient characteristics and clinical observation

**FIGURE 1:**
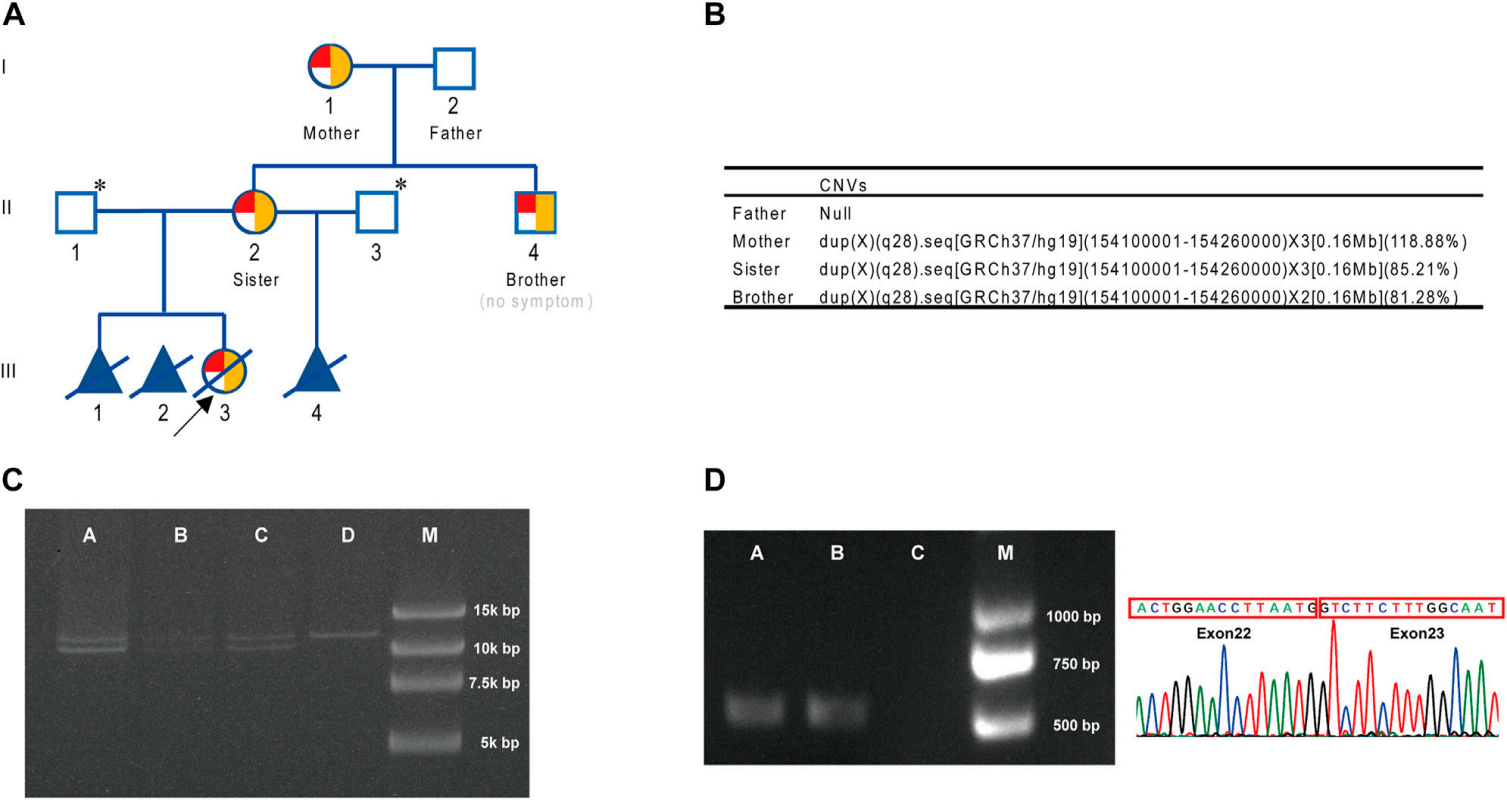
Pedigree and genetic analysis. **(A)** Genogram for the family (with CNV and inversion results). Red for inversion, 1/4 red circle for heterozygosis, 1/2 red circle for homozygosis, and yellow for duplication. ^*^ indicates that *F8* gene analysis was not performed. **(B)** CNV results for the family. **(C)** Inv22 assay for the family. A, brother (II-4); B, sister (II-2); C, mother (I-1); D, father (I-2); M, marker. **(D)** Connection between exon 22 and exon 23 at the transcript level. A, brother (II-4); B, sister (II-2); C, blank; M, marker.

In a missed abortion of a woman (II-2) (Figure 1A), chromosomal microarray analysis (CMA) of aborted tissue (III-3, female fetus) was performed in other hospital, and duplication of Xq28 (arr [GRCh37] Xq28 (154109413–154228924) ×3) was found. As this region involved *F8*, the relatively high incidence of *F8* inversion was further analyzed, and Inv22 was also found in the aborted tissue. Then, the woman (II-2) came to our hospital for genetic counseling on the reproductive risk of hemophilia A. As there was no hemophilia A patient in her family, we further analyzed the peripheral blood samples from the woman (II-2) by CMA and the *F8*-specific inversion panel. Also, we found duplication in the Xq28 region (arr [GRCh37] Xq28 (154120633–154228924) ×3) and Inv22. Because Inv22 is a common pathogenic variant of hemophilia A, the woman (II-2) preferred to perform genetic testing of her family to assess the pathogenicity of this complex variant. Blood coagulation factor VIII activities in all four family members (I-1, I-2, II-2, and II-4) were normal (Supplementary Material S3).

### Genetic testing

CNV analysis was conducted on the four members of this family, and it was found that his mother (I-1), sister (II-2), and the male subject (II-4) all carried a 0.16 Mb duplication of *F8* gene ([GRCh37/hg19] chr.X: 154100001–154260000) (Figure 1B). The duplication region contained exon 1–exon 22 of *F8* gene and partial of its flanking sequences (including partial intron 22 and 5′-UTR of *F8*). Inv22 analysis of the family members using the *F8*-specific inversion panel revealed that the mother (I-1) and the woman (II-2) both carried heterozygous Inv22, and her younger brother (II-4) was hemizygous, while her father (I-2) was normal (Figure 1C). Interestingly, the woman‘s younger brother (II-4) also carried this Inv22 of *F8*. At that time, he was a college student of 24 years old who usually liked playing basketball and never experienced joint swelling and pain, gingival bleeding, or unstoppable bleeding. That is to say he did not have a significant hemophilia A disease phenotype (blood coagulation factor VIII activities 69.1% (Supplementary Material S3)).

### Gene expression

In general, Inv22 of the *F8* gene in males always results in an absent normal transcript, that is, there is no exon 22–exon 23-connected transcript (exon 23 is located after the breakpoint of the inversion). However, there were exon 22–exon 23-linked transcripts in this male (II-4) (Figure 1D), that is, there were normal *F8* gene transcripts in this male. This result suggested that Inv22 might be located in the duplication region and did not affect the normal expression of *F8*.

**FIGURE 2:**
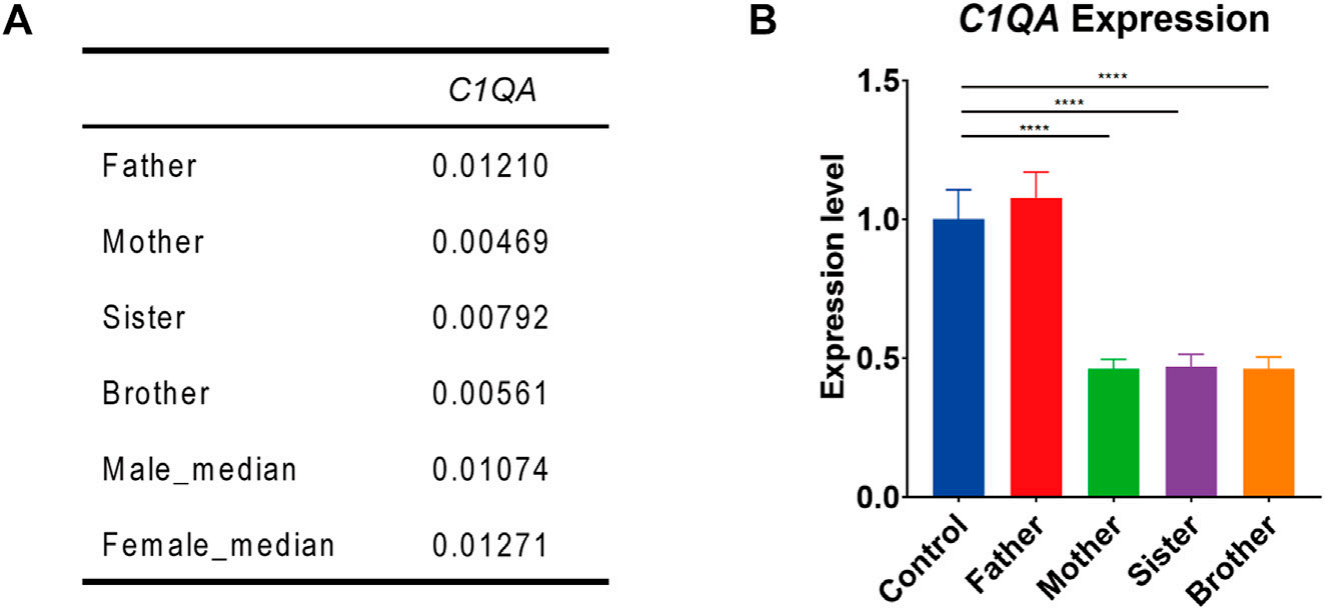
*C1QA* expression in the family members. **(A)** *C1QA* expression levels in four family members and reference databases (male_median and female_median) by RNA-seq analysis (values represented the relative expression to *GAPDH*). **(B)**. Q-PCR confirmation of *C1QA* expression in four family members and normal control (one-way ANOVA and Dunnett’s multiple comparison test; ^****^, *p* < 0.0001).

RNA-seq analysis showed that the expression of *F8* was very low in the blood of all the family members. But interestingly, the expression of *C1QA* in this male (II-4) as well as in his mother (I-1) and sister (II-2) was only half of that in his father (I-2) and normal control. This was also validated by Q-PCR (Figure 2).

## Discussion

**FIGURE 3:**
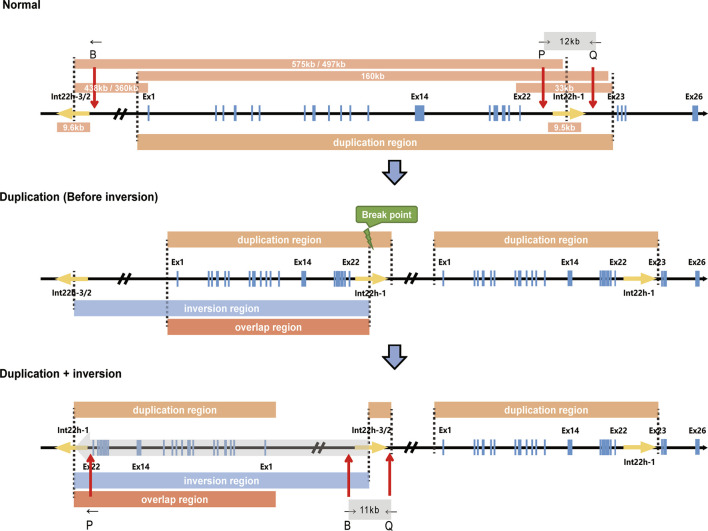
Schematic description of *F8* duplication and inversion. The upper, middle, and lower panels represent the normal *F8*, the duplication of *F8* (in which inversion has not occurred yet), and the duplication and further inversion of *F8*, respectively. The size of specific regions, duplication regions, inversion regions, and regions overlapping duplication and inversion are indicated. B, P, and Q show the relative positions of the primers used in Inv22 analysis. Ex1, Exon 1; Ex14, Exon 14; Ex22, Exon 22; Ex23, Exon 23; Ex26, Exon 26.

In this case, we presented a male without obvious hemophilia A phenotype but hemizygous for complex Inv22 of the *F8* gene, accompanied by a partial duplication of 0.16 Mb. This partial duplication and Inv22 in *F8* were also found in his mother and sister. Considering that the exon 22–exon 23-linked transcripts and Inv22 both presented in the male (II-4), combined with the mechanism by which Inv22 occurs, we speculated that Inv22 occurred in the duplicated region, which was likely to be located upstream of *F8* (Figure 3). By analyzing the RNA expression, we found that the expression of *C1QA* in his mother, sister, and the male subject was about only half of that in his father and normal population. This was the first report of such a complex structural variation of *F8* in an asymptomatic male.

The most common variant of *F8* is Inv22 (Antonarakis et al., 1995), and the remaining variants include Inv1, point mutations (including nonsense mutations), and small deletions (Bagnall et al., 2002; Oldenburg et al., 2004). In 2,401 patients with hemophilia type A (mild, moderate, and severe), the incidence of large duplications (>50bp) was about 0.46% (11/2,401), compared with Inv22 (24.1%), Inv1 (0.83%), missense mutation (46.4%), frameshift mutation (10.2%), nonsense mutation (6.2%), and splicing mutation (3.0%) (Johnsen et al., 2017). So far, only 52 HA patients with large duplications of *F8* have been reported (Table 1). Specifically, six of these patients were combined with *F8* inversion. Two patients (one male and one female) with Inv22, combined with exon 23–25 duplication, had no overlap between the inversion and the duplication region (Johnsen et al., 2017). One severe HA patient had overlapped duplication (intron 1–6) and inversion region (intron 1) in *F8* (Sanna et al., 2013). Only Lannoy et al. (2015) reported similar inversion duplication within *F8* intron 1 from three unrelated families with mild hemophilia A. In general, Inv1 of the *F8* gene always caused severe hemophilia A, but in those probands, tandem inversion duplication (210 kb) did not completely disrupt the *F8* expression and still retained a small number of normal mRNA transcripts. In contrast to mild hemophilia A, we reported that the FVIII factor activity reached 69.1% (normal range 70%–150%) in the asymptomatic male (II-4) with duplication and Inv22. Therefore, it was speculated that this structural variation not only brought no changes to the integrity of the open reading frame but also had no significant effect on the promoter of *F8*.

**TABLE 1:** Fifty-two HA patients with large duplications of *F8*.

Number	PMID	Reference	Gender	Severity	Duplication			Inversion	
					Variant exon/intron	Duplicated exon	size (kb)	Variant exon/intron	size (kb)
1	23140572	SANNA et al. (2013)	M	Severe hemophilia A	Intron 1–6	Exon 2–6	19.32	Int1h	—
2	25962585	Lannoy et al. (2015)	M	Mild hemophilia A	*F8 Intron 1/VBP1 Intron 4*	Exon 1	210	Int1h	—
3			M	Mild hemophilia A	*F8 Intron 1/VBP1 Intron 4*	Exon 1	210	Int1h	—
4			F	Mild hemophilia A	*F8 Intron 1/VBP1 Intron 4*	Exon 1	210	Int1h	—
5	29296726	Johnsen et al. (2017)	M	—	Exon 23–25	Exon 23–25	—	Int22 h	—
6			F	—	Exon 23–25	Exon 23–25	—	Int22 h	—
7			—	—	Exon 2	Exon 2	—		
8			—	—	Exon 2–4	Exon 2–4	—		
9			—	—	Exon 4–22	Exon 4–22	—		
10			—	—	Exon 5–22	Exon 5–22	—		
11			—	—	Exon 6	Exon 6	—		
12			—	—	Exon 6–26	Exon 6–26	—		
13			—	—	Exon 13	Exon 13	—		
14			—	—	Exon 14	Exon 14	—		
15			—	—	Exon 19–22	Exon 19–22	—		
16			—	—	Exon 22	Exon 22	—		
17			—	—	Exon 23–25	Exon 23–25	—		
18	18752578	Rost et al. (2008)	M	Mild hemophilia A	Exon 13	Exon 13	—		
19			—	Severe hemophilia A	Exon 14	Exon 14	—		
20			—	Severe hemophilia A	Exon 1–5	Exon 1–5	—		
21			—	Severe hemophilia A	Exon 5–25	Exon 5–25	—		
22			—	Severe hemophilia A	Exon 23–25	Exon 23–25	—		
23			—	Severe hemophilia A	Exon 23–25	Exon 23–25	—		
24			—	Severe hemophilia A	Exon 2–25	Exon 2–25	—		
25			—	Severe hemophilia A	Exon 14–21	Exon 14–21	—		
26			—	Severe hemophilia A	Exon 7–11	Exon 7–11	—		
27	34964972	Wang et al. (2012)	M	Severe hemophilia A	Exon 1–22	Exon 1–22	—		
28	23299923	Lannoy et al. (2013)	M	Moderate hemophilia A	Intron 22-Int22h-2	Exon 1–22	500		
					Intron 1–14	Exon 2–14	82		
29			M	Severe hemophilia A	Int22h-1–Int22h-3	Exon 1–22	600		
30	31445452	Lannoy et al. (2019)	M	Unknown (fetus)	Exon 1–13	Exon 1–13	75		
31	23140572	Sanna et al. (2013)	M	Severe hemophilia A	Int1h-1–Intron 6	Exon 2–6	19.32		
32	2105106	Casula et al. (1990)	—	Mild hemophilia A	Exon 13	Exon 13	—		
33	28475226	Johnsen et al. (2017)	M	Severe hemophilia A	Intron14-Int1h-2	Exon 1–14	231		
					Intron 1–Exon 11 (triplication)	Exon 2–11	52.5		
34			M	Severe hemophilia A	*F8 Intron3-TMLHE Intron 3*	Exon 133	498		
35			M	Severe hemophilia A	*F8*	Exon 1 he	1302		
					*F8 (3′extremity to Exon 22) (Triplication*)	Exon 22ati	233		
36	https://doi.org/10.1182/blood.V110.11.1149.1149	Vinciguerra et al. (2007)	—	Severe hemophilia A	Intron 10–14	Exon 11–14	—		
37	22621702	Vencesla et al. (2012)	M	Severe hemophilia A	Exon 2–10	Exon 2–10	—		
38			M	Severe hemophilia A	Exon 14 and Exon 23–25	Exon 14 and Exon 23–25	—		
39	22103590	Miller et al. (2012)	M	—	Exon 2–20	Exon 2–20	—		
40	20735723	Zimmermann et al. (2010)	M	Severe hemophilia A	Exon 14–21	Exon 14–21	—		
41			M	Severe hemophilia A	Exon 7–11	Exon 7–11	—		
42			M	Mild hemophilia A	Exon 14	Exon 14	—		
43			M	Severe hemophilia A	Exon 13	Exon 13	—		
44			M	Severe hemophilia A	Exon 1–5	Exon 1–5	—		
45			M	Severe hemophilia A	Exon 5–25	Exon 5–25	—		
46			M	Severe hemophilia A	Exon 23–25	Exon 23–25	—		
47			M	Severe hemophilia A	Exon 2–25	Exon 2–25	—		
48			M	Severe hemophilia A	Exon 23–25	Exon 23–25	—		
49			M	Severe hemophilia A	Exon 22	Exon 22	—		
50			M	Severe hemophilia A	Exon 7–22	Exon 7–22	—		
51			M	Severe hemophilia A	Exon 23–26	Exon 23–26	—		
52	21371190	Rafati et al. (2011)	M	Severe hemophilia A	Exon 24	Exon 24	—		

Values in the eighth column refer to the sizes of the duplication regions (kb).

C1qA is the A-chain peptide of C1q, which binds C1r and C1s to produce the first component of the serum complement system. The complement system is the humoral backbone of innate immune defense and is involved in many different processes, including antibacterial defense, clearance of immune complexes, and tissue regeneration. C1, the identifying unit of the complement system, is a complex with a size of 790 kDa, which contains three subcomponents: C1q, C1r, and C1s. C1q is the recognition component, and C1r and C1s are two proteases. The two copies of C1r and C1s combine to form a Ca^2+^-dependent tetramer, which together with C1q forms C1 (Gaboriaud et al., 2004). *C1QA* is a 3,216-bp gene and locates on chromosome 1, encoding 245 aa (NM_015991.4). It is widely expressed in the spleen, lymph node, whole blood, and 29 other tissues (https://www.genecards.org/cgi-bin/carddisp.pl?gene=C1QA&keywords=C1QA#expression). C1qA is associated with angiogenesis, innate immune response, osteosarcoma (OS), schizophrenia, hypertension, aging, and obesity. According to rat aortic ring assay, C1q induced permeability of the endothelial cell (EC) monolayer, stimulated proliferation and migration of the EC, and promoted tube formation and the sprouting of new vessels (Bossi et al., 2014). In innate immune response, C1qA interacted with various components of the RIG-I (retinoic acid-inducible gene-I) pathway and enhanced the RIG-I-VISA (virus-induced signaling adaptor)-mediated signaling pathway and TBK1 (TANK-binding kinase 1)-mediated activation of the interferon-β promoter (Wang et al., 2012). Rarely, people with a deficiency of the complement component C1q were prone to recurrent infections with polysaccharide-containing encapsulated microorganisms and a high prevalence of autoimmune diseases, mainly systemic lupus erythematosus (SLE) (Sun-Tan et al., 2010; Hayakawa et al., 2011). *C1QA*, *C1QB*, and *C1QC* expression levels were positively correlated with OS patient survival time and negatively correlated with the clinicopathological feature percent necrosis at definitive surgery (Chen et al., 2021). In schizophrenia, the gene expression of complement pathway activators (C1qA) and mediators (C3 or C4) was increased in the midbrain, especially in patients with inflammatory biotypes (Purves-Tyson et al., 2020). The loss of *C1QA* would also reduce hypertension-induced β-catenin signaling, proliferation of vascular smooth muscle cells, and pathological arterial remodeling (Sumida et al., 2015). During normal aging, the levels of the C1q protein in the mouse and human brain dramatically increased, and in certain tests of hippocampus-dependent behavior, C1q-deficient mice showed no cognitive or memory decline compared to their wild-type littermates (Stephan et al., 2013; Soto et al., 2015). In the diet-induced obesity mouse model, C1qA was necessary to cause damage to cerebral vasculature and white matter (Graham et al., 2020).

The expression of *C1QA* in this male (II-4) as well as in his mother (I-1) and sister (II-2) was only half of that in his father (I-2) and normal control. It is unknown whether this change in *C1QA* expression is directly related to the structural variation of *F8*, but we speculate that it may be at some risk in wound healing, recurrent infections, and even autoimmune diseases, but whether there will be related symptoms requires further follow-up.

The limitation to this study was the regulatory mechanisms of *C1QA*. The impact of *F8* on *C1QA* expression and its association with this complex structural variant also required further investigation in animal models and cell models.

In conclusion, here, we presented an asymptomatic male with complex hemizygous Inv22 of the *F8* gene, accompanied by a partial duplication of 0.16 Mb. This partial duplication and Inv22 in *F8* were also found in his mother and sister. By RNA-seq analysis, we also found that the expression of *C1QA* in his mother, sister, and the male subject was about only half of that in his father and normal population. As far as we know, this is the first report of partial duplication and Inv22 in *F8* in a male without hemophilia A phenotype. Therefore, caution is recommended in the use of conventional Inv22 or Inv1 assay for clinical prediction, especially with no family history of hemophilia A. Lastly, our study highlighted the complexity of the underlying molecular mechanisms in *F8* structural variation in hemophilia A.

## Data Availability

The RNA-seq data presented in the study are deposited in the Genome Sequence Archive for human repository (https://ngdc.cncb.ac.cn/gsa-human/), accession number HRA003790. The data of chromosomal microarray analysis presented in the study are deposited in the OMIX repository (https://ngdc.cncb.ac.cn/omix/), accession number OMIX002784.

## Supplementary material

Supplementary Material S1 The values of the read counts (RNA-seq) after normalization using DESeq2.

Supplementary Material S2 RNA-seq data analysis codes.

Supplementary Material S3 Biochemical tests results of coagulation factor VIII.
