# Supplementary material for: F8 gene inversion and duplication cause no obvious hemophilia A phenotype
Source: Front Genet. 2023 Feb 9;14:1098795. doi: 10.3389/fgene.2023.1098795 (PMC9947239; doi:10.3389/fgene.2023.1098795)
Supplement: Supplementary file 1 [file Table2.docx]

#FastQC

fastqc -t 8 in.R1.fq.gz in.R2.fq.gz -o output dir

#cutadapt

cutadapt -j 10 --minimum-length 30 -a AGATCGGAAGAGCACACGTCTGAACTCCAGTCAC -A AGATCGGAAGAGCGTCGTGTAGGGAAAGAGTGT -o R1_cutadapt.fastq.gz -p R2_cutadapt.fastq.gz R1.fq.gz R2.fq.gz

#alignment

STAR --runMode genomeGenerate --genomeDir genomeDir --genomeFastaFiles hg19.fasta --runThreadN 12

STAR --twopassMode Basic --genomeDir genomeDir --runThreadN 12 --outSAMtype BAM SortedByCoordinate --twopass1readsN -1 --sjdbOverhang 75 --readFilesIn R1_cutadapt.fastq.gz R2_cutadapt.fastq.gz --readFilesCommand zcat --outSAMattrRGline ID:RNAseq SM:LCL10488 PL:ILLUMINA

mv Aligned.sortedByCoord.out.bam sorted.bam

samtools rmdup sorted.bam deduped.bam

RNASplitReadsAtJunction --reassign_mapq 255:60 -r hg19.fasta -i deduped.bam splitted.bam

#Count reads

htseq-count -r name -I gene_name -f bam splitted.bam Homo_sapiens.GRCh37.87.gtf > sampleid.counts

#Difference analysis

Library(‘DEGseq’)

library(dplyr)

DEG_screen <- function(counts1,count2, ref_median_counts, ref_mean_counts,){

counts1_counts2_DEG<-DEGexp(geneExpMatrix1 = counts1, geneCol1 = 1, expCol1 = 2, geneExpMatrix2 = counts2, geneCol2 = 1, expCol2 = 2, pValue = 0.05, zScore = 4, qValue = 0.01 foldChange = 1, thresholdKind = 5, normalMethod = 'none', method = 'MARS', outputDir = outdir)

counts1_median_DEG<-DEGexp(geneExpMatrix1 = counts1, geneCol1 = 1, expCol1 = 2, geneExpMatrix2 = ref_median_counts, geneCol2 = 1, expCol2 = 2, pValue = 0.05, zScore = 4, qValue = 0.01 foldChange = 1, thresholdKind = 5, normalMethod = 'none', method = 'MARS', outputDir = outdir)

counts1_mean_DEG<-DEGexp(geneExpMatrix1 = counts1, geneCol1 = 1, expCol1 = 2, , geneExpMatrix2 = ref_mean_counts, geneCol2 = 1, expCol2 = 2, pValue = 0.05, zScore = 4, qValue = 0.01 foldChange = 1, thresholdKind = 5, normalMethod = 'none', method = 'MARS', outputDir = outdir)

counts2_median_DEG<-DEGexp(geneExpMatrix1 = counts2, geneCol1 = 1, expCol1 = 2, geneExpMatrix2 = ref_median_counts, geneCol2 = 1, expCol2 = 2, pValue = 0.05, zScore = 4, qValue = 0.01 foldChange = 1, thresholdKind = 5, normalMethod = 'none', method = 'MARS', outputDir = outdir)

counts2_mean_DEG<-DEGexp(geneExpMatrix1 = counts2, geneCol1 = 1, expCol1 = 2, , geneExpMatrix2 = ref_mean_counts, geneCol2 = 1, expCol2 = 2, pValue = 0.05, zScore = 4, qValue = 0.01 foldChange = 1, thresholdKind = 5, normalMethod = 'none', method = 'MARS', outputDir = outdir)

intersect<-inner_join(counts1_counts2_DEG, counts1_median_DEG, counts1_mean_DEG, counts2_median_DEG , counts2_mean_DEG ,by="gene_name")

return(intersect)

}

Son_counts <- read.csv(file = Son_countsfile, sep = '\t', header = T, row.names = 1)

Daughter_counts <- read.csv(file = Daughter_countsfile, sep = '\t', header = T, row.names = 1)

Father_counts <- read.csv(file = Father_countsfile, sep = '\t', header = T, row.names = 1)

Mather_counts <- read.csv(file = Mather_countsfile, sep = '\t', header = T, row.names = 1)

Male_median_counts <- read.csv(file = Male_median_countsfile, sep = '\t', header = T, row.names = 1)

Male_mean_counts <- read.csv(file = Male_mean_countsfile, sep = '\t', header = T, row.names = 1)

Female_median_counts <- read.csv(file = Female_median _countsfile, sep = '\t', header = T, row.names = 1)

Female_mean_counts <- read.csv(file = Female_mean_countsfile, sep = '\t', header = T, row.names = 1)

Son_ intersect <- DEG_screen(Son_counts, Father_counts, Male_median_counts, Male_mean_counts)

Daughter_ intersect <- DEG_screen(Daughter _counts, Mather_counts, Female_median_counts, Female_mean_counts)

All_intersect<- inner_join(Son_ intersect, Daughter_ intersect,by=’ gene_name’)

write.table(All_intersect,file="All_intersect.xls",sep="\t",row.names=F,quote=F)

#Create ref_database

python3 miRNATargetGenePredict.py --exprdir Expression -m Homo_sapiens_GRCh37_87_chr_patch_hapl_scaff_id_name.mapping --log log.txt

python3 miRNATargetGeneEnrichment.py --exprdir Expression -gm gomapping.tmp --species hsa --keggclass kegg_name.class --bg Homo_sapiens_GRCh38_101_chr_patch_hapl_scaff.keggbackground --anno Homo_sapiens_GRCh38_101_chr_patch_hapl_scaff.anno --log log.txt --go go.class

python3 miRNADiffExpr.py --exprdir Expression --cond train_set_condition.txt --cmp cmp.txt --log log.txt -n 3 -p 0.05 -q 0.01 -fc 1.5 --de_method 1 --newcond train_set_newcondition.txt --newcmp train_set_newcmp.txt --degseqcond train_set_degseq_condition.txt --degseqcmp train_set_degseq_cmp.txt

#Count normalization

library(ggplot2)

library(RColorBrewer)

library(DESeq2)

library(pheatmap)

## Load in data

data <- read.table("data/full_counts.txt", header=T, row.names=1)

meta <- read.table("meta/full_meta.txt", header=T, row.names=1)

### Check classes of the data we just brought in

class(data)

class(meta)

### Check that sample names match in both files

all(names(data) %in% rownames(meta))

all(names(data) == rownames(meta))

## Create DESeqDataset Object

dds <- DESeqDataSetFromMatrix(countData = round(data), colData = meta, design = ~ sampletype)

#View(counts(dds))

dds <- estimateSizeFactors(dds)

normalized_counts <- counts(dds, normalized = TRUE)

write.table(normalized_counts, file="data/chenwei_normalized_counts.txt", sep="\t", quote=F, col.names=NA)
