# Supplementary material for: F8 gene inversion and duplication cause no obvious hemophilia A phenotype
Source: Front Genet. 2023 Feb 9;14:1098795. doi: 10.3389/fgene.2023.1098795 (PMC9947239; doi:10.3389/fgene.2023.1098795)
Supplement: Supplementary file 2 [file Table3.docx]

**Supplementary Material (S2):** **Biochemical tests results**

| **Biochemical tests** | **Father** | **Mother** | **Sister** | **Brother** | **Unit** | **reference range** |
| --- | --- | --- | --- | --- | --- | --- |
| Activity of Coagulation Factor VIII | 101.2 | 89.4 | 72.9 | 69.1 | % | 70-150 |
